# Associations between self-reported obstetric complications and experience of care: a secondary analysis of survey data from Ghana, Kenya, and India

**DOI:** 10.1186/s12978-022-01546-z

**Published:** 2023-01-06

**Authors:** Ntemena Kapula, Emma Sacks, Dee T. Wang, Osamuedeme Odiase, Jennifer Requejo, Patience A. Afulani, the Revisioning EmONC Quality of Care Workgroup, Lenka Benova, Andreea Creanga, Louise Tina Day, Lynn Freedman, Kathleen Hill, Allison Morgan, Sodzi Sodzi-Tettey, Dilys Walker, Catherine Breen, Jean Pierre Monet, Allisyn Moran, Moise Muzigaba, Blerta Maliqi, Ozge Tuncalp, Tedbabe Hailegebriel

**Affiliations:** 1 Department of Epidemiology and Biostatistics, University of California, San Francisco, 550 16th St, San Francisco, CA 94158 USA; 2 Division of Data Analytics, Planning and Monitoring, United Nations Children’s Fund, New York, NY USA; 3 Mailman School of Public Health-Population and Family Health, Columbia University, New York, NY USA; 4 Institute for Global Health Sciences, University of California, San Francisco, San Francisco, CA USA

**Keywords:** Experience of care, Obstetric complications, India, Kenya, Ghana, c-Section

## Abstract

**Background:**

Although several indicators have been proposed to measure women’s experience of care in health facilities during the intrapartum period, it is unknown if these indicators perform differently in the context of obstetric emergencies. We examined the relationship between experience of care indicators from the Person-Centered Maternity Care (PCMC) scale and obstetric complications.

**Methods:**

We used data from four cross-sectional surveys conducted in Kenya (rural: N = 873; urban: N = 531), Ghana (N = 531), and India (N = 2018) between August 2016 and October 2017. The pooled sample included 3953 women aged 15–49 years who gave birth within 9 weeks prior to the survey. Experience of care was measured using the PCMC scale. Univariate, bivariate, and multivariable analyses were conducted to examine the associations between the composite and 31 individual PCMC indicators with (1) obstetric complications; (2) severity of complications; and (3) delivery by cesarean section (c-section).

**Results:**

16% (632) of women in the pooled sample reported obstetric complications; and 4% (132) reported having given birth via c-Sect. (10.5% among those with complications). The average standardized PCMC scores (range 0–100) were 63.5 (SD = 14.1) for the full scale, 43.2 (SD = 20.6) for communication and autonomy, 67.8 (SD = 14.1) for supportive care, and 80.1 (SD = 18.2) for dignity and respect sub-scales. Women with complications had higher communication and autonomy scores (45.6 [SD = 20.2]) on average compared to those without complications (42.7 [SD = 20.6]) (p < 0.001), but lower supportive care scores, and about the same scores for dignity and respect and for the overall PCMC. 18 out of 31 experience of care indicators showed statistically significant differences by complications, but the magnitudes of the differences were generally small, and the direction of the associations were inconsistent. In general, women who delivered by c-section reported better experiences.

**Conclusions:**

There is insufficient evidence based on our analysis to suggest that women with obstetric complications report consistently better or worse experiences of care than women without. Women with complications appear to experience better care on some indicators and worse care on others. More studies are needed to understand the relationship between obstetric complications and women’s experience of care and to explore why women who deliver by c-section may report better experience of care.

**Supplementary Information:**

The online version contains supplementary material available at 10.1186/s12978-022-01546-z.

## Plain language summary

In several studies and reports, women have described mistreatment by health providers during childbirth in health facilities. Particularly in low- and middle-income countries, such mistreatment has negative effects on women’s decisions to seek maternity care in health facilities. It is unclear if women with complications are more or less likely to experience some forms of mistreatment compared to women without complications. In this study, we examined 31 experience of care indicators in three domains: (1) Supportive Care; (2) Respect and Dignity; and (3) Communication and Autonomy from the validated Person-Centered Maternity Care (PCMC) questionnaire. We compare these experience of care indicators between women who report obstetric complications and those who don’t report complications, by the reported severity of the complications, and by their mode of delivery. The study included data from three countries: Ghana, Kenya, and India. The results showed that the experience of care among women who reported obstetric complications was not consistently better or worse than that of those who did not have complications. Therefore, efforts should be made to improve the experience of care in health facilities for every birthing woman. Additionally, women who delivered via c-section had consistently better experiences than women who delivered vaginally. More studies are needed to understand the relationship between mode of delivery and women’s experience of care.

**Supplementary Information:**

The online version contains supplementary material available at 10.1186/s12978-022-01546-z.

## Introduction

Person-centered maternity care (PCMC) refers to care that is respectful and responsive to the needs and values of individual women and their families during childbirth [1, 2]. PCMC is a universal human right and essential for a safer and more positive childbirth experience [3]. It is also a key component of quality of care and critical to improving maternal and neonatal outcomes [4, 5]. The key elements of PCMC include supportive care, communication and autonomy, and dignity and respect [6]. Despite efforts to improve women’s experience of care in many settings, poor experience of care remains a persistent problem during childbirth [6, 7].

Over the past decade, there has been a growing body of research examining the role of poor experience of care, mistreatment, and lack of support in facility-based childbirths [8–10]. Prior studies have shown that improving women’s experience of care in health facilities during and immediately after childbirth may improve maternal and neonatal health outcomes [4, 11, 12], and increase the likelihood that women will choose to deliver in a health facility in future pregnancies [8, 13].

Although several quantitative studies have examined women’s experience of care (inclusive of respectful maternity care, mistreatment, and disrespect and abuse) during childbirth in health facilities, very few have examined differences in experience of care in the context of obstetric complications [7, 9, 14]. Raj et al. created a composite measure to examine the association between mistreatment by health providers during childbirth and maternal health complications in India and found that women who reported mistreatment by providers were more likely to have maternal complications at delivery (adjusted odds ratio = 1.32; 95% CI: 1.05–1.67) [15]. A study in Tanzania found that women who reported most types of complications during childbirth were more likely to report disrespect and abuse while those who delivered by c-section were less likely to report any disrespect and abuse [16]. In both studies, participants reporting yes on any one of several items on mistreatment or disrespect and abuse were viewed as having experienced mistreatment/disrespect and abuse by a provider during childbirth. In a prior analysis from Ghana, Kenya and India using the single composite PCMC score, there was no significant difference in PCMC scores by pregnancy complications [6, 17]. However, this association was not examined for the individual indicators or domains that comprise the PCMC score.

As part of the process to review and revise the global Emergency Obstetric and Newborn Care (EmONC) framework [18], our group was tasked with identifying experience of care indicators that might be most relevant to women experiencing obstetric complications. To help inform this process, we conducted a secondary analysis of available datasets including both complications and an experience of care measure, to assess whether women who self-report obstetric complications are more likely to report negative or positive experiences of care during childbirth. We identified four datasets from three countries: Kenya, Ghana, and India. These surveys used the validated PCMC scale that is comprised of 30 experience of care indicators spanning three domains, (1) supportive care; (2) communication and autonomy; and (3) dignity and respect. These surveys also asked questions about complications in a standardized way. This secondary analysis will contribute to the evidence base on the linkages between obstetric complications and experience of care.

## Methods

### Data sources

The datasets used for this secondary analysis are from four different cross-sectional surveys administered in three countries: one from rural Ghana (N = 531), two from Kenya (rural: N = 873, urban: N = 531)[1], and one from rural India (N = 2018). Details of the original studies and data collection for the surveys are described in a previous publication and presented in Additional file 1: Appendix S1 [6]. Briefly, each survey was conducted with women aged 15–49 years who had recently given birth (within 9 weeks prior to the survey); surveys were conducted by trained research assistants after discharge from the facility, either in a private area at the health facility, or later in the woman’s home. Each survey utilized the 30-item PCMC Scale, with some surveys including additional questions on women’s experience of care during childbirth.

The survey in Ghana was conducted in five health facilities in East Mamprusi district in northern Ghana with the goal of obtaining data for the evaluation of an intervention to improve quality of maternal and newborn care [19]. Although 588 women completed the survey, our analysis is based on responses from the 531 women who had complete information on the 30 PCMC items and obstetric complication questions.

Two surveys were conducted in Kenya. One survey was conducted in Migori County located in rural western Kenya as part of a research study on community perceptions of the quality of care during childbirth [1]. 1052 women completed this survey, but our analysis is based on the 873 women with complete data on all relevant variables. The other survey from Kenya was conducted in Nairobi and Kiambu Counties, which are considered urban, to obtain baseline data for the evaluation of an intervention to improve person-centered care [20]. Analyses were performed on responses from all the women who responded to the survey (N = 531).

In India*,* the survey was conducted in 40 public health facilities across 20 districts in the State of Uttar Pradesh as part of a study on quality of maternity care [21]. Analyses were performed on responses from all the women who participated in the survey (N = 2018).

The interviews were conducted in multiple local languages in each setting (Additional file 1: Appendix S1). Women provided individual consent to participate. Ethical approval for the original studies were obtained from the Institutional Review Boards of the Kenya Medical Research Institute, the Navrongo Health Research Center in Ghana, the Community Empowerment Lab in India, and the University of California, San Francisco.

### Measures

*Experience* *of* *care* *indicators*. The experience of care indicators used in this analysis are from the PCMC scale [1, 6, 22], which is an interviewer-administered questionnaire with 3 sub-scales for (1) supportive care, (2) communication and autonomy, and (3) dignity and respect. The original scale, which was validated in Kenya, includes 30-items, with an additional question on bribes in the Indian version of the scale. In addition, all surveys except the Indian survey included eight additional experience of care indicators which are also examined (Table 2).

### Obstetric complications

Obstetric complications were captured in three ways (1) self-reported experience of a complication during pregnancy or perinatal period; (2) self-reported severity of the complication; and (3) mode of delivery (vaginal vs c-section).

*Obstetric* *complications* is a binary variable derived from a survey question asking: “At any time during labor, delivery, or after delivery did you suffer from any health problems? (yes/no)” Women who responded *“yes”* were asked to specify what health problems they had, thus ensuring all problems reported were related to either pregnancy or childbirth. The list of complications reported is shown in Additional file 1: Appendix S2. All datasets contained this question.

The *severity* *of* *obstetric* *complications* *variable* combined responses from women in rural Ghana and rural Kenya responding positively to the above question about having an obstetric complications and responding to a follow-up question about severity: “Will you say this problem was severe?” The respondents that responded “no” to the first question were coded “0, No complications”, those that responded “yes” to the first question and *“no”* to the second were coded “1, Mild complication” and those that responded *“yes”* to both questions were coded “2, Severe complication”. Data on severity of obstetric complications was not collected in urban Kenya and India.

*Mode* *of* *delivery*: The mode of delivery variable was measured by one question; “Was your baby delivered by cesarean section?” and the response options were “no” or “yes” or “don’t know”. The “don’t know” response options were recoded to “no”. This variable was included in the analysis because it may be a more objective way to capture which women experienced obstetric emergencies, but we recognize that it includes women who may have had c-sections for non-emergency reasons. Only the surveys from India, Ghana and rural Kenya included this question.

*Covariates.* Age, parity, marital status, education, employment, household wealth, antenatal complications, facility type, and gender of provider were examined as covariates based on prior research findings of association with experience of care. All variables used in this analysis were self-reported and have been described in detail elsewhere [6].

### Statistical analysis

All data were imported into STATA 17.0 for analysis [23]. The four datasets were aggregated to create a pooled sample of respondents to obtain a large enough sample to increase the stability of estimates among women reporting complications (given the relatively small proportion of women reporting complications, presented in the results). Descriptive statistics were conducted on each of the datasets as well as for the pooled sample. Mean scores were calculated for each experience of care indicator, ranging from 0 to 3 (higher values depicting more positive experiences), and summative scores generated for the full PCMC scale and for the three subscales. Bivariate analyses involved examining the unadjusted associations between each experience of care indicator and summative scores by—(1) reported obstetric complication, (2) reported severity, and (3) reported mode of delivery—using two-sample t-tests and one-way ANOVA. Finally, we conducted multivariable linear regression analysis with robust standard errors of the PCMC and subscale scores on obstetric complications and mode of delivery, controlling for relevant covariates [10]. Given the cross-sectional nature of the data and potential reverse causality, we also conducted a secondary analysis with c-section as the outcome, using multivariable logistic regression.

## Results

### Univariate results

#### Characteristics of sample

Table 1 shows the demographic characteristics of the women in the pooled sample. Most of the women were aged between 15 and 22 years, had a parity of two, were married, had no school or primary education only, and were not formally employed. About 16% (N = 645) reported having obstetric complications, with the highest percentage of complications in the Ghana sample (36%) and the lowest complication rate in the India sample at 11% (see Additional file 1: Appendix S3 for the distribution of study variables by country). About 4% (N = 132) of women from Ghana, rural Kenya and India reported giving birth via c-section (9% 8%, and 1% respectively). As expected, women with complications were more likely to have a c-Sect. (10.5% for those with a complication compared to 2.5% for those without a complication). Among the 132 women who gave birth via c-section, 45% reported having a complication (Additional file 1: Appendix S5).

**Table 1:** Characteristics of women in pooled sample from Kenya, Ghana and India, N = 3953

	n (%)
*Country/* *setting*	
Rural Kenya	873 (22%)
Urban Kenya	531 (13%)
Ghana	531 (13%)
India	2018 (51%)
*Age*	
15 to 22 years	1299 (33%)
23 to 25 years	963 (24%)
26 to 28 years	748 (19%)
29 to 48 years	942 (24%)
*Parity* *(no.* *of* *prior* *births)*	
0–1	612 (19%)
2	1101 (34%)
3	748 (23%)
4 +	782 (24%)
*Marital* *status*	
Single	211 (5%)
Partnered/cohabiting	109 (3%)
Married	3569 (90%)
Widowed/ divorced/separated	64 (2%)
*Education*	
No school or primary	2383 (60%)
Post primary, vocational, or secondary	1109 (28%)
University/ college or above	461 (12%)
*Employed*	
No	3307 (84%)
Yes	646 (16%)
*Household* *wealth* *quintile*	
Poorest	727 (19%)
Poor	756 (19%)
Middle	738 (19%)
Rich	694 (18%)
Richest	1023 (26%)
*Had* *pregnancy* *complications*	
No	1598 (40%)
Yes	2355 (60%)
*Delivery* *facility* *type*	
Government Hospital	1603 (41%)
Government Health Center	2010 (51%)
Mission or private facility	331 (8%)
*Delivery* *provider* *gender*	
Male	451 (11%)
Female	3368 (85%)
Both	124 (3%)
*Obstetric* *complications*	
No obstetric complications	3308 (84%)
Obstetric complications	645 (16%)
*Mode* *of* *delivery* (n = 3419)*	
Vaginal delivery	3287 (96%)
C-section	132 (4%)

Data are n (%)

Characteristics of women by country are in Additional file 1: Appendix S2

*Does not include data from urban Kenya

#### Distributions of experience of care indicators

Table 2 shows the distributions of the experience of care indicators in the pooled sample. The distributions by country are shown in Additional file 1: Appendix S4 and have also been reported elsewhere [6]. In the supportive care domain, one third of respondents reported that they were never allowed to have a companion of choice during delivery and providers never talked to them about their feelings. In the communication and autonomy domain, more than 80% of respondents reported that providers did not introduce themselves at the first encounter. More than half reported that providers did not ask for consent before performing procedures on them. About 50% of the women reported that providers did not explain the exams or procedures performed on them and over 40% reported that providers did not explain the medicines they were prescribed. In the dignity and respect domain, approximately 53% of respondents felt they were treated with respect all the time. About 16% reported verbal abuse and 4% reported physical abuse at least once during their stay at the health facility.

**Table 2:** Distribution of responses on the experience of care indicators, Total n = 3953

EOC indicators	No, never n (%)	Yes, a few times n (%)	Yes, most of the time n (%)	Yes, all the time n (%)
*Supportive* *care:* *mean* *summative* *score:* *67.8* *(SD = 14.1)* ^*d*^				
Time to care^a^	223 (6%)	465 (12%)	1057 (27%)	2208 (56%)
Labor support	663 (17%)	333 (8%)	634 (16%)	2323 (59%)
Delivery support	1327 (34%)	236 (6%)	433 (11%)	1957 (50%)
Able to talk about feelings	1169 (30%)	1311 (33%)	873 (22%)	600 (15%)
Received support when anxious	909 (23%)	1090 (28%)	769 (20%)	1185 (30%)
Received attention when needed help	207 (5%)	746 (19%)	1362 (35%)	1638 (41%)
Provider took best care	113 (3%)	670 (17%)	1596 (40%)	1574 (40%)
Pain control	828 (21%)	827 (21%)	1219 (31%)	1079 (27%)
Trust in health providers	110 (3%)	364 (9%)	1111 (28%)	2368 (60%)
Enough staff present	232 (6%)	656 (17%)	1293 (33%)	1772 (45%)
Crowded facility*	594 (15%)	858 (22%)	1264 (32%)	1237 (31%)
Clean environment^b^	357 (9%)	504 (13%)	1661 (42%)	1431 (36%)
Water present at facility	317 (8%)	224 (6%)	741 (19%)	2671 (68%)
Electricity present at facility	58 (2%)	250 (6%)	1359 (34%)	2286 (58%)
Felt safe at facility	64 (2%)	179 (5%)	703 (18%)	3007 (76%)
*Communication* *and* *autonomy:* *mean* *summative* *score:* *43.2* *(SD = 20.6)*^*d*^				
Provider introduced self^c^	3481 (88%)	255 (7%)	126 (3%)	91 (2%)
Called by name	1192 (30%)	857 (22%)	688 (17%)	1216 (31%)
Involvement in care	1482 (38%)	626 (16%)	608 (15%)	1237 (31%)
Consented to procedures	2113 (54%)	569 (14%)	607 (15%)	664 (17%)
Delivery position choice	1402 (36%)	905 (21%)	705 (18%)	941 (24%)
Providers spoke language you understood	55 (1%)	436 (11%)	1940 (49%)	1522 (39%)
Provider explained exams/ procedures	1946 (49%)	642 (16%)	633 (16%)	732 (19%)
Provider explained medicines	1601 (49%)	680(17%)	598 (15%)	1074 (27%)
Able to ask questions	755 (19%)	869 (22%)	1007 (26%)	1322 (33%)
*Dignity* *and* *respect:* *mean* *summative* *score:* *80.1* *(SD = 18.2)*^*d*^				
Treated with respect	198 (5%)	540 (14%)	1117 (28%)	2098 (53%)
Health staff Friendly	163 (4%)	625 (16%)	1154 (29%)	2011 (51%)
Verbal abuse*	3304 (84%)	394 (10%)	183 (5%)	72 (2%)
Physical abuse*	3811 (96%)	82 (2%)	44 (1%)	16 (0.4%)
Visual privacy	922 (23%)	251 (6%)	512 (13%)	2268 (57%)
confidentiality of records	408 (10%)	672 (17%)	912 (23%)	1961 (50%)
*Additional* *EoC* *indicators*				
Asked for a bribe*	3141 (80%)	616 (16%)	172 (4%)	24 (0.6%)
Providers showed care**	57 (3%)	235 (12%)	674 (35%)	969 (50%)
Privacy during discussions**	1203 (62%)	277 (14%)	231 (12%)	224 (12%)
Providers ask about pain**	474 (25%)	467 (24%)	490 (25%)	504 (26%)
Received attention during stay at facility**	95 (5%)	340 (18%)	740 (38%)	760 (39%)
Allowed to eat and drink**	399 (21%)	382 (20%)	444 (23%)	710 (38%)
Patient forced to stay against their will due to lack of pay**	1848 (96%)	50 (3%)	25 (1%)	12 (1%)
Patient treated differently because of personal attributes**	1833 (95%)	57 (3%)	27 (1%)	18 (1%)

^a^Time to care: Very long, somewhat long, somewhat short, very short

^b^Clean environment: Very dirty, dirty, clean, very clean

^c^Introduce self-responses: No, none of them; Yes, a few of them; Yes, most of them; Yes, all of them

^d^Summative scores generated by adding up responses to all items in the subscale and standardized to range from 0 to 100

*Responses were reversed during analysis so that the higher the code value the more positive the experience

**Does not include data from India (n = 1935)

The average standardized PCMC score for the pooled sample was 63.5 (SD = 14.1) out of 100, where 0 is the worst PCMC score and 100 is the best. PCMC scores for the Ghana sample at 63.2 (SD = 15.8), rural Kenya 65.6 (SD = 15.5), urban Kenya 66.6 (SD = 13.4) and India 61 (SD = 12.9). The lowest score in the pooled sample was from the communication and autonomy domain at 43.2 (SD = 20.6), followed by supportive care domain score of 67.8 (SD = 14.1), and the dignity and respect domain score at 80.1 (SD = 18.2).

### Bivariate results

#### Experience of care indicators by obstetric complications

Table 3 shows the results of the bivariate analyses of the experience of care indicators by reported obstetric complications for the pooled dataset (N = 3953). Mean scores for each experience of care indicator, as well as differences in the mean scores for women who reported obstetric complications and those who did not report complications are reported. Of the 31 experience of care indicators analyzed in the pooled sample, 18 showed statistically significant differences by complications (Box 1). The differences in mean scores among variables with statistically significant associations were, however, generally small (defined as <|0.25|), except for the following indicators that had an absolute difference of at least 0.25 and above: delivery support (− 0.59), consented to procedures (0.26), delivery position choice (− 0.26), visual privacy (0.27), and medical record confidentiality (0.26) (Table 3).

**Table 3:** Bivariate analysis of experience of care indicators by obstetric complication in pooled sample (n = 3953)

Experience of care indicators	Obstetric complications (n = 632)	No obstetric complications (n = 3321)		
	(a) Mean score (SD)	(b) Mean score (SD)	(a-b) Difference of mean scores	P-value
*Supportive* *care*	66.6 (14.4)	68.0 (14.0)	**− 1.4**	**0.02**
Time to care	2.27 (0.98)	2.34 (0.87)	− 0.07	0.09
Labor support	2.02 (1.22)	2.20 (1.13)	− 0.17	** < 0.001**
Delivery support	1.27 (1.38)	1.86 (1.33)	− **0.59**	** < 0.001**
Talked to about feelings	1.35 (1.08)	1.20 (1.02)	0.15	**0.00**
Support anxiety	1.51 (1.18)	1.57 (1.14)	− 0.06	0.24
Attention when need help	2.09 (0.96)	2.13 (0.88)	− 0.04	0.33
Provider took best care	2.19 (0.87)	2.17 (0.80)	0.02	0.59
Control pain	1.72 (1.11)	1.63 (1.09)	0.09	0.06
Trust in provider	2.38 (0.85)	2.47 (0.76)	− 0.09	**0.01**
Enough staff present	2.11 (0.93)	2.18 (0.90)	− 0.06	0.08
Crowded facility	1.69 (1.09)	1.78 (1.00)	− 0.09	0.05
Clean environment	2.06 (0.85)	2.05 (0.93)	0.00	0.79
Water at facility	2.43 (0.91)	2.46 (0.92)	− 0.04	0.45
Electricity at facility	2.55 (0.72)	2.47 (0.67)	0.08	**0.01**
Felt safe at facility	2.61 (0.73)	2.70 (0.61)	− 0.09	**0.00**
*Communication* *and* *autonomy*	45.6 (20.2)	42.7 (20.6)	**2.9**	**0.00**
Providers introduce self	0.26 (0.67)	0.19 (0.59)	0.07	**0.01**
Patient called by name	1.56 (1.20)	1.47 (1.21)	0.08	0.09
Involvement in care	1.47 (1.24)	1.39 (1.28)	0.08	0.14
Consent to procedures	1.17 (1.26)	0.91 (1.14)	**0.26**	** < 0.001**
Delivery position choice	1.08 (1.19)	1.34 (1.17)	− **0.26**	** < 0.001**
Provider used language patient understood	2.38 (0.76)	2.22 (0.69)	0.16	** < 0.001**
Explain exams/ procedures	1.20 (1.20)	1.01 (1.17)	0.19	** < 0.001**
Explain medicines	1.29 (1.24)	1.09 (1.20)	0.20	**0.00**
Able to ask questions	1.59 (1.16)	1.76 (1.11)	− 0.17	** < 0.001**
*Dignity* *and* *respect*	81.1 (18.1)	79.9 (18.2)	**1.2**	0.12
Treated with respect	2.29 (0.88)	2.29 (0.88)	− 0.01	1.00
Friendly	2.22 (0.92)	2.28 (0.86)	− 0.05	0.13
Verbal abuse	2.64 (0.77)	2.77 (0.59)	− 0.13	** < 0.001**
Physical abuse	2.88 (0.47)	2.96 (0.27)	− 0.07	** < 0.001**
Visual privacy (were covered)	2.27 (1.14)	2.00 (1.27)	**0.27**	** < 0.001**
Record confidentiality	2.34 (0.96)	2.08 (1.04)	**0.26**	** < 0.001**
*Additional* *indicators*				
Bribe	2.68 (0.64)	2.75 (0.55)	0.05	**0.01**

Bold = statistically significant (p < 0.05) differences and differences greater than 0.25

The direction of the associations also differed across the indicators (summarized in Box 1). Of the five indicators with differences greater than 0.25, women with obstetric complications reported lower scores on delivery support and delivery position of choice. Women with complications reported higher scores on consent to procedures, visual privacy, and medical record confidentiality. In general, women with obstetric complications had higher mean scores on most indicators in the communication and autonomy domain and lower mean scores on most indicators in the supportive care domain. The average communication and autonomy domain score was 3 points higher for women with complications (45.6 [SD = 20.2]) than for those without any complications (42.7 [SD = 20.6]) (p < 0.001). In contrast, the average supportive care domain score was lower for women who reported obstetric complications (66.6[SD = 14.4]) than for women without complications (68.0[SD = 14.0]) (p = 0.02). For dignity and respect, women with complications appeared to have better privacy and confidentiality but were more likely to experience verbal and physical abuse statistically significant. Women with complications were less likely to be asked for a bribe.

Box 1. A comparison of experience of care indicators by obstetric complications*

**Table:** 

Experience of care domain	Experience of care indicators: better when obstetric complications reported	Experience of care indicators: worse when obstetric complications reported
**Supportive care domain**	Able to talk about feelings Electricity present at the facility	Labor support **Delivery support** Trust in providers Felt safe at facility
**Communication and autonomy domain**	Providers introduced themselves **Consent to procedures** Language Provider explained exams/ procedures Provider explained medicines	**Delivery position of choice** Able to ask provider questions
**Dignity and respect domain**	**Visual privacy** **Confidentiality of records**	Verbal abuse Physical abuse
**Additional experience of care indicator**	Asked for bribe	

*These indicators include only those for which statistically significant (p < 0.05) differences were found. Bolded have magnitude difference greater or equal to |0.25|

#### Experience of care indicators by severity of obstetric complications

Table 4 shows the differences of mean scores among women with varying levels of self-reported severity of obstetric complications (no obstetric complications, mild obstetric complications, or severe obstetric complications) from Ghana and rural Kenya (differences in mean scores by level of severity shown in Additional file 1: Appendix S6). There were no substantial differences by severity of complications, and the direction of association was not consistent. In general, women with severe complications had lower mean scores on most of the supportive care indicators than those with mild or no complications, with large statistically significant differences (i.e. magnitudes greater than 0.25) for delivery support and pain control. Women with mild or severe complications reported lower delivery support scores than those with no complications, and better pain control was reported among those with mild complications compared to those with no complications. For communication and autonomy, women with severe complications reported higher scores on being called by name and providers explaining the purpose of exams and procedures, but worse scores on being involved in their care, consenting for exams and procedures, and providers explaining medicines than those with mild and no complications. Although several indicators in the dignity and respect domain had significant differences, only confidentiality of records was substantially different with higher scores reported among those with a mild complication compared to those with no complication. Women who reported severe complications also reported better scores on being allowed to eat or drink than those that did not report any complications.

**Table 4:** Mean scores of experience of care indicators by severe obstetric complications in Ghana and Rural Kenya, n = 1404

	Mean scores (SD) of women with no complications (n = 1058)	Mean scores (SD) of women with mild complications (n = 110)	Mean scores (SD) of women with severe complications (n = 236)	P-value
*Supportive* *care,* *mean* *(SD)*	68.0 (14.1)	66.2 (14.6)	64.3 (15.8)	**0.00**
Time to care	2.33 (0.87)	2.36 (0.86)	2.16 (1.08)	**0.04**
Labor support	1.88 (1.12)	1.75 (1.14)	1.96 (1.23)	0.28
Delivery support	0.81 (1.14)	0.45 (0.87)	0.50 (0.97)	**0.00**
Talked about feeling	1.61 (1.05)	1.66 (1.04)	1.54 (1.14)	0.55
Support anxiety	1.64 (1.16)	1.66 (1.17)	1.51 (1.21)	0.28
Attention when need help	2.09 (0.90)	2.25 (0.84)	2.04 (1.06)	0.14
Took best care	2.34 (0.74)	2.39 (0.69)	2.25 (0.94)	0.19
Control pain	1.41 (1.18)	1.82 (1.20)	1.58 (1.18)	**0.00**
Trust	2.33 (0.77)	2.4 (0.78)	2.31 (0.92)	0.61
Enough staff present	2.05 (1.01)	2.01 (0.93)	2.09 (1.01)	0.77
Crowded	1.90 (1.10)	1.86 (1.04)	1.71 (1.11)	0.06
Clean environment	2.02 (0.47)	1.95 (0.30)	2.05 (0.44)	0.16
Water at facility	2.45 (0.79)	2.42 (0.75)	2.36 (0.90)	0.30
Electricity at facility	2.47 (0.78)	2.64 (0.62)	2.56 (0.84)	**0.04**
Felt safe at facility	2.50 (0.72)	2.56 (0.76)	2.53 (0.78)	0.64
*Communication* *and* *autonomy*	42.7 (20.6)	47.9 (22.8)	47.1 (22.1)	**0.00**
Introduce self	0.46 (0.87)	0.45 (0.84)	0.34 (0.77)	0.15
Called by name	1.57 (1.23)	1.44 (1.15)	1.75 (1.27)	0.05
Involvement in care	1.81 (1.17)	1.66 (1.10)	1.56 (1.24)	**0.01**
Consent to procedures	1.54 (1.22)	1.79 (1.22)	1.44 (1.30)	0.05
Delivery position choice	0.77 (1.06)	0.94 (1.14)	0.80 (1.12)	0.28
Language	2.52 (0.74)	2.6 (0.68)	2.53 (0.86)	0.57
Explain exams/ procedures	1.55 (1.20)	1.17 (1.20)	1.45 (1.23)	**0.01**
Explain medicines	1.65 (1.20)	1.54 (1.30)	1.34 (1.27)	**0.00**
Able to ask questions	1.50 (1.14)	1.36 (1.10)	1.37 (1.23)	0.18
*Dignity* *and* *respect* *domain*	79.9 (18.2)	82.8 (18.0)	82.5 (18.6)	**0.03**
Treated with respect	2.37 (0.79)	2.15 (0.85)	2.33 (0.86)	**0.03**
Friendly	2.33 (0.81)	2.15 (0.86)	2.28 (0.92)	0.09
Verbal abuse	2.79 (0.61)	2.66 (0.79)	2.63 (0.88)	**0.00**
Physical abuse	2.93 (0.36)	2.88 (0.48)	2.82 (0.60)	**0.00**
Visual privacy (were covered)	2.28 (1.09)	2.44 (0.93)	2.33 (1.07)	0.30
Record confidentiality	2.29 (0.86)	2.6 (0.77)	2.44 (0.87)	**0.00**
*Additional* *experience* *of* *care* *indicators*				
Bribe	2.90 (0.41)	2.89 (0.41)	2.80 (0.54)	**0.01**
Lack of privacy during discussions	2.14 (1.10)	2.08 (1.15)	2.26 (1.07)	0.24
Providers ask about pain	1.51 (1.12)	1.69 (1.11)	1.62 (1.19)	0.15
attention during stay at facility	2.16 (0.84)	2.27 (0.81)	2.09 (0.99)	0.19
Allowed to eat and drink	1.46 (1.16)	1.67 (1.17)	1.75 (1.19)	**0.00**
Patient forced to stay	2.91 (0.40)	2.93 (0.40)	2.90 (0.43)	0.81
Patient treated differently	2.90 (0.44)	2.95 (0.27)	2.83 (0.60)	**0.04**
Providers showed care	2.34 (0.79)	2.31 (0.83)	2.35 (0.83)	0.91

Bold = statistically significant (p < 0.05) differences

#### Experience of care indicators by mode of delivery (c-section/vaginal delivery)

Table 5 shows the bivariate analysis results of PCMC and domain scores by mode of delivery from women in Rural Kenya, Ghana, and India. Women who delivered via c-section reported better scores on most indicators compared to women who delivered vaginally. For supportive care, compared to women who delivered vaginally, women who delivered via c-section reported better scores on being able to talk about feelings, receiving support for anxiety, pain control, and having enough staff. For communication and autonomy, compared to women who delivered vaginally, women who delivered via c-section reported better scores on being called by name, being involved in their own care, giving consent to procedures, providers explaining exams/ procedures, and ability to ask provider questions. Providers were more likely to ask about pain to women who had a c-section than to those who delivered vaginally. The magnitude of the difference with these variables were all greater than 0.25 but none greater than 1. None of the dignity and respect variables had statistically significant associations except for visual privacy. The mean PCMC and domain scores, on average, were higher for women who gave birth via c-section at 68.4 (SD = 15.3) compared to those that gave birth vaginally at 62.9 (SD = 14.1). The differences of mean PCMC and domain scores by the mode of delivery were statistically significant except for the supportive care domain.

**Table 5:** Mean scores of experience of care by mode of delivery in pooled sample (Rural Kenya, Ghana and India) n = 3419

	C-Section, N = 132 Mean (SD)	Vaginal Delivery, N = 3287 Mean score (SD)	Difference between mean scores	p-value
*Supportive* *care* *domain*^1^	68.3 (SD = 14.6)	68.2 (SD = 14.2)	0.1	0.96
Time to care	2.26 (1.05)	2.31 (0.89)	− 0.05	0.61
Labor support	1.91 (1.22)	1.88 (1.15)	0.03	0.79
Delivery support	0.68 (1.14)	0.75 (1.13)	− 0.07	0.54
Talk about feeling	1.86 (1.08)	1.57 (1.05)	**0.29**	**0.01**
Support Anxiety	1.94 (1.21)	1.70 (1.33)	0.24	**0.04**
Attention when need help	2.26 (0.90)	2.07 (0.93)	0.18	**0.03**
Took best care	2.48 (0.70)	2.31 (0.78)	0.17	**0.01**
Control pain	1.75 (1.11)	1.44 (1.18)	**0.32**	**0.00**
Trust	2.50 (0.66)	2.31 (0.81)	0.19	**0.00**
Enough staff present	2.35 (0.87)	2.01 (1.01)	**0.33**	** < 0.001**
Crowded	1.81 (1.06)	1.89 (1.10)	− 0.07	0.43
Clean environment	2.15 (0.65)	2.17 (0.60)	− 0.02	0.74
Water present at facility	2.59 (0.69)	2.40 (0.82)	0.19	**0.00**
Electricity present at facility	2.52 (0.82)	2.49 (0.77)	0.04	0.70
Felt safe at facility	2.58 (0.69)	2.49 (0.74)	0.09	0.16
*Communication* *and* *autonomy* *domain*^*1*^	55.7 (SD = 22.2)	40.6 (SD = 20.0)	15.1	**0.00**
Introduce self	0.54 (1.00)	0.42 (0.84)	0.12	0.18
Called by name	1.85 (1.25)	1.55 (1.22)	**0.30**	**0.01**
Involvement in care	2.08 (1.15)	1.78 (1.27)	**0.30**	**0.01**
Consent to procedures	1.89 (1.17)	1.50 (1.23)	**0.39**	** < 0.001**
Delivery position choice	0.82 (1.12)	0.78 (1.07)	0.04	0.70
Language	2.48 (0.83)	2.52 (0.75)	− 0.04	0.70
Explain exams/ procedures	1.88 (1.19)	1.46 (1.21)	**0.42**	** < 0.001**
Explain medicines	1.85 (1.32)	1.79 (1.28)	0.06	0.63
Able to ask questions	1.75 (1.21)	1.43 (1.14)	**0.32**	**0.01**
*Dignity* *and* *respect* *domain*^*1*^	84.7 (SD = 16.4)	80.0 (SD = 18.5)	4.7	**0.00**
Treated with respect	2.39 (0.88)	2.33 (0.82)	0.06	0.46
Friendly	2.29 (0.94)	2.30 (0.84)	0.00	0.91
Verbal abuse	2.73 (0.73)	2.76 (0.67)	− 0.04	0.66
Physical abuse	2.82 (0.54)	2.91 (0.41)	− 0.09	0.07
Visual privacy (were covered)	2.38 (0.73)	2.59 (0.72)	− 0.22	**0.00**
Record confidentiality	2.42 (0.84)	2.32 (0.87)	0.10	0.21
*Additional* *experience* *of* *care* *indicators*				
Bribe	2.86 (0.47)	2.88 (0.44)	− 0.02	0.65
Lack of privacy during discussions**	2.12 (1.20)	2.16 (1.09)	− 0.04	0.72
Providers ask about pain**	1.84 (1.10)	1.51 (1.13)	**0.33**	**0.00**
Received attention during stay at facility**	2.29 (0.76)	2.14 (0.88)	0.15	**0.04**
Allowed to eat and drink**	1.35 (1.18)	1.54 (1.165)	− 0.19	0.09
Patient forced to stay**	2.73 (0.73)	2.93 (0.35)	− 0.20	**0.00**
Patient treated differently**	2.78 (0.67)	2.90 (0.44)	− 0.12	0.05
Providers showed care**	2.48 (0.76)	2.32 (0.81)	0.16	**0.03**

Does not include data from Urban Kenya

Bolded p-value is below 0.05

^**^Does not include data from India (n = 1401)

^1^Summative scores generated by adding up responses to all items in the subscale/domain

Box 2. A comparison of experience of care indicators by mode of delivery*

**Table:** 

Experience of care domain	Experience of care indicators: better when c-section delivery reported	Experience of care indicators: worse when c-section delivery reported
**Supportive care domain**	**Able to talk about feelings** Support anxiety Attention when need help Took best care **Control pain** Trust in providers **Enough staff present** Water present at delivery facility	
**Communication and autonomy domain**	**Called by name** **Involved in care** **Consent to procedures** **Explain exams/procedures** **Able to ask questions**	
**Dignity and respect domain**		Visual privacy
**Additional experience of care indicator**	**Providers ask about pain** Received attention during stay at delivery facility Providers showed they cared	Patient forced to stay in delivery facility

*These indicators include only those for which statistically significant (p < 0.05) differences were found. Bolded indicators have a magnitude difference greater or equal to |0.25|

### Multivariable results

Table 6 shows multivariable linear regression of PCMC and domain scores by obstetric complications, mode of delivery, and selected covariates. Holding other factors constant, giving birth via c-section was associated with a higher PCMC score (β = 4.36 [95% CI: 1.56–7.16]) than giving birth vaginally, but there was no significant difference by obstetric complication. Of note however, when the analysis was stratified by country (Additional file 1: Appendix S7), while women who delivered via c-section in the rural Kenya sample had higher mean PCMC scores (β = 8.22 [95%CI:4.82, 11.6]), women who delivered by c-section in the India sample had lower mean PCMC scores (β = − 7.57 [95%CI: − 9.98, − 5.16]), with no significant difference in the Ghana sample. Also, women with complications in the rural Kenya sample had lower PCMC scores (β = − 3.24 [95% CI: − 6.09, − 0.40]), but no statistically significant difference in the other samples. In the model using sub-scales, giving birth via c-section was associated with a higher communication and autonomy score (β = 7.87 [95% CI: 3.82,11.9]) and a higher supportive care score (β = 4.06 [95% CI: 1.18, 6.95]). Independent of other factors, the odds of giving birth via c-section was associated with a higher PCMC score (OR:1.02; 95%CI: 1.01–1.03) and higher communication and autonomy scores (OR: 1.02; 95%CI: 1.00–1.03) (Additional file 1: Appendix S8).

**Table 6:** Multivariate linear regression model of PCMC and PCMC domains on obstetric complications (Rural Kenya, Ghana, and India), n = 2687

	PCMC	Supportive care	Communication and autonomy	Dignity and respect
	Coef. [robust std. err.]	Coef. [robust std. err.]	Coef. [robust std. err.]	Coef. [robust std. err.]
*Obstetric* *complications*				
No obstetric complications	Ref.			
Obstetric complications	1.13 [− 2.72, 0.46]	− 1.12 [− 2.73,0.49]	− 1.39 [− 3.65, 0.87]	− 0.71 [− 2.64, 1.23]
*Mode* *of* *delivery*				
Vaginal delivery	Ref.			
Cesarean delivery	4.36** [1.56, 7.16]	4.06** [1.18, 6.95]	7.87*** [3.82, 11.9]	0.22 [− 2.91, 3.36]
Constant	58.8*** [55.9, 61.8]	60.6*** [57.6, 63.6]	40.6*** [36.4, 44.8]	78.4*** [75.0, 81.8]

Controlling for age, parity, marital status, education, paid employment, household wealth, pregnancy complications, provider gender, country

Full table available upon request

Missing data in some covariates hence reduced sample size

*p < 0.05, **p < 0.01, ***p < 0.001

## Discussion

The objective of this study was to assess if there are substantial differences in experiences of care between women with or without obstetric complications. We pooled data on women who had recently given birth in Ghana, Kenya, and India from surveys that included the PCMC tool. The analysis showed that, although there were statistically significant differences in women’s experiences by complications based on several indicators, the magnitudes of these differences were in general small, and the directions of the associations were inconsistent. Based on summative sub-scale scores for the pooled sample, women who had complications had, on average, higher communication and autonomy scores, but lower supportive care scores, and about the same scores for dignity and respect and for the overall PCMC compared to women without complications. The findings were similar when we examined the association between experience of care and severity of complications. The direction of association was more consistent for mode of delivery for the pooled sample, with women who delivered via c-section reporting higher overall PCMC scores compared to those who delivered vaginally, and higher communication and autonomy and supportive care scores. However, there were variations by country, including in the direction of associations.

The lack of an overall association between PCMC scores and presence of an obstetric complication is inconsistent with previous research, where differences have been identified [6, 17, 19, 21]. But the lower PCMC scores among women with obstetric complications for the rural Kenya sample (in the stratified analysis) is consistent with prior studies. Findings from a study in Uttar Pradesh, India, for example, found that women who reported mistreatment by a provider during childbirth had higher odds of complications at delivery and postpartum [15]. Similarly, a study in Tanzania found that women who reported any complication during childbirth and those with self-reported depression in the last year were more likely to report any disrespect and abuse, while those who delivered by c-section were less likely to report any disrespect and abuse [16]. A longitudinal study in Kenya also found that women who reported higher PCMC scores at delivery had significantly lower risk of reporting both maternal and newborn complications and screening positive for depression at 2 and 10 weeks postpartum [4, 11]. This is the first study, however, to examine in detail the association between different experience of care indicators and domains from the PCMC scale with self-reported obstetric complications, severity of the complications, and mode of delivery. The inconsistency in the direction of associations for the individual indicators by complication explains the lack of significant association based on the overall PCMC scores. It also highlights that associations with composite scores will depend on the specific items constituting the score, stressing the importance of standardized tools. Provider interactions with patients may be influenced by the type and severity of the complication, which is a potential reason for the difference in the findings for the measure of complications and c-section. Further, the influence of receipt of c-section on experience may be capturing other factors such as the ability to get elective c-sections, which is influenced by social status. Moreover, the differences in the direction of the associations by country suggest contextual differences in how women with complications and those who deliver via c-section may be treated. These findings are important for understanding how women’s experiences may differ when they have a complication in a given context and for guiding future research.

Although the magnitude of the differences was small, women who reported a complication reported better experiences on several items in the communication and autonomy domain, which recorded the lowest mean score in the univariate analysis. Women with complications were more likely to report that providers introduced themselves, used a language they could understand, explained the purpose of exams/procedures and medicines, and asked for consent, than those without complications. On the other hand, women with complications were less likely to be allowed a birthing position of choice and more likely to report that they did not feel they could ask providers any questions. Potential reasons for these findings are that women with complications may be seen by specialists or unfamiliar providers, who recognize the need to introduce themselves, yet these providers might not realize that women with complications may have questions about their immediate care. Providers handling complex cases may also feel a greater need to properly inform and get consent from patients with complications because of the potential fear of litigation for adverse outcomes [24]. Another potential reason for improved communication reported by women with complications is that women with complications may stay longer in health facilities resulting in increased interaction with health providers, which in this case appeared more positive. Women who give birth by c-section are more likely to have longer stays in health facilities [25], which might explain their better experiences. In the survey in Tanzania, women who stayed less than a day in the facility were more likely to report disrespect and abuse, while women who delivered by c-section were less likely to report any disrespect and abuse [16].

In the supportive care domain, women who reported obstetric complications were more likely to report that providers talked to them about how they were feeling but they were less likely to be allowed a companion during labor and delivery. Women who had c-sections additionally reported better experiences compared to women who delivered vaginally, specifically on providers supporting them with their anxieties, paying attention when they needed help, and providing them with options for pain control. This difference could be because there is a general increase in provider attention when women have complications, [26] but women with complications may also be isolated from their families and other support persons leading to a lower sense of safety. For example, in a previous study in Kenya, providers reported not allowing companions for women with complications because they believed the presence of companions interfered with clinical management of the woman [27]. Women with complications are also more likely to give birth in higher-level facilities, where they might be less familiar, feel less safe, and be less trustful of providers [21].

In general, most women reported being treated with dignity and respect, regardless of complications. Women with complications were, however, more likely to report better privacy and feeling their medical records were kept confidential. Potential reasons include providers taking extra efforts to prevent other patients from seeing records related to the management of complications. Certain complications may also be treated in more private areas in the hospital. On the other hand, previous research suggests verbal and physical abuse tend to be heightened when there is a complication, the sense of urgency is high, and providers are afraid of a poor outcome, which might explain the higher reporting of verbal and physical abuse among women with complications in the study settings [28, 29]. Reports of more verbal and physical abuse were consistent with findings from two prior studies that examined women’s experiences and certain types of complications, using measures focused on disrespect and abuse [12, 13].

### Limitations and strengths

There are some limitations in our study. Firstly, the four datasets used were designed for different purposes and not specifically to examine differences in experience of care by obstetric complication. Secondly, all data including obstetric complications are self-reported and subject to recall and social desirability bias. Studies using observation and medical record review may more accurately capture complications but may need to be combined with surveys to understand women’s experiences. Thirdly, all the surveys were cross-sectional, and we are unable to assess the temporal order of experiences and onset of complications or indication for c-section. Longitudinal or observational studies that account for temporal ordering of events are needed to assess associations between women’s experiences and complications. Larger studies may also be needed to better capture experiences within a particular location, given the small sample of women with complications in a given setting. The proportion of births that are complicated varies for different sub-populations, especially based on age and parity, and estimates of complication rates from various studies vary ranging from about 15 to 23% of which 8% are considered life threatening [30–32]. Qualitative studies to complement quantitative analyses would also provide a more in-depth understanding of women’s preferences and experiences during complications. We were unable to examine differences by specific complications, but rather we examined differences based on self-assessed severity of the complication and mode of delivery. This was further limited by the fact that questions were not asked consistently across the surveys, and we were, therefore, unable to analyze differences by severity of complications and mode of delivery for all the settings. All the datasets were obtained by non-probability sampling methods and are not nationally representative; the different sample sizes also imply the findings may be influenced more by the largest samples (e.g., India). We were also limited by the locations in which there were existing datasets that included both complication data and an experience of care score. Thus, there are limitations with regards to generalizability within and across the study settings.

This study has several strengths. The experience of care indicators are from a validated scale and questions had been examined in cognitive interviews to assess their relevance and comprehensibility in the study settings. The questions are also comprehensive—beyond mistreatment or disrespect and abuse—to capture the three domains of experience of care from the WHO vision for quality of maternal and newborn health [6]. Examining individual experience of care indicators enabled us to tease out the different aspects of care that were influenced by the presence of an obstetric complication. We were able to use multi-country data, providing sufficient sample size for country stratification. Using data from different countries collected from the same tool helps to increase the applicability of the findings in other settings. The findings provide preliminary information into how women’s experiences may differ in multiple settings for women with and without obstetric complications.

## Conclusion

To our knowledge, this is the first attempt at analyzing in detail the association between individual experience of care indicators with obstetric complications. We found that reporting on several indicators differed between women with and without obstetric complications, but the direction of the association was inconsistent. Given the small magnitude of the differences in various indicators by complications, there is not sufficient evidence to suggest that women with obstetric complications have markedly different experiences overall than women without. In fact, women with complications may experience better communication than those with no complications. On the other hand, women with complications may be more likely to experience verbal and physical abuse and not be accompanied by a birth companion and may desire more labor support and options. Programs promoting the prevention of verbal and physical abuse, promotion of birth companionship and increased patient choice, could therefore be emphasized in interventions targeting women with complications, within the context of improving respectful care for everyone. Quality improvements for everyone (including those without complications) should aim to improve communication, consent, privacy, and other aspects of respectful care.

While more studies (both qualitative and quantitative) are needed to better understand women’s experience of care in the context of obstetric complications and mode of delivery, given the generally low PCMC scores for both women with and without complications, and the critical importance of respectful and responsive care, interventions to improve the experience of care for all women are urgently needed.

## Supplementary Information

**Additional file 1: Appendix S1.** Datasets from Ghana, Rural Kenya, Urban Kenya, and India; total n = 3,953; August 2016 – October 2017. **Appendix S2.** Survey questions capturing reported obstetric complications**. Appendix S3**. **Characteristics of women from rural Kenya, urban Kenya, Ghana and India, N=3,953. Appendix S4.** Distribution of experience of care indicators in four datasets, N = 3,953. **Appendix S5. **2x2 table of mode of delivery by obstetric complications, N = 3419 (row totals).** Appendix S6.** Difference in mean scores of experience of care indicators by severe obstetric complications in Ghana and Rural Kenya, N=1,404.** Appendix S7.** Multivariate Linear regression of PCMC on selected covariates in Kenya, Ghana, and India. **Appendix S8.** Multivariable logistic regression models of C-section on PCMC and PCMC domains (rural Kenya, Ghana and India), n =2687.

## Data Availability

The data analyzed for this manuscript are available from the corresponding author on reasonable request.

## Abbreviations

PCMC: Person Centered Maternity Care
WHO: World Health Organization
C-section: Cesarean section
UCSF: University of California, San Francisco
KEMRI: Kenya Medical Research Institute (Kenya)
IPA: Innovations for Poverty Action (Kenya)
NHRC: Navrongo Health Research Center (Ghana)
CEL: Community Empowerment Lab (India)
