# Supplementary material for: Associations between self-reported obstetric complications and experience of care: a secondary analysis of survey data from Ghana, Kenya, and India
Source: Reprod Health. 2023 Jan 6;20:7. doi: 10.1186/s12978-022-01546-z (PMC9817240; doi:10.1186/s12978-022-01546-z)
Supplement: Supplementary file 1 — Additional file 1: Appendix S1. Datasets from Ghana, Rural Kenya, Urban Kenya, and India; total n = 3,953; August 2016 – October 2017. Appendix S2. Survey questions capturing reported obstetric complications. Appendix S3. Characteristics of women from rural Kenya, urban Kenya, Ghana and India, N=3,953. Appendix S4. Distribution of experience of care indicators in four datasets, N = 3,953. Appendix S5. 2x2 table of mode of delivery by obstetric complications, N = 3419 (row totals). Appendix S6. Difference in mean scores of experience of care indicators by severe obstetric complications in Ghana and Rural Kenya, N=1,404. Appendix S7. Multivariate Linear regression of PCMC on selected covariates in Kenya, Ghana, and India. Appendix S8. Multivariable logistic regression models of C-section on PCMC and PCMC domains (rural Kenya, Ghana and India), n =2687. [file 12978_2022_1546_MOESM1_ESM.docx]

**Additional file 1**

**Appendix S1.**

| **Datasets from Ghana, Rural Kenya, Urban Kenya, and India; total n = 3,953; August 2016 – October 2017** | | | | |
| --- | --- | --- | --- | --- |
|  | **Ghana** | **Rural Kenya** | **Urban Kenya** | **India** |
| **N** | 531 | 873 | 531 | 2,018 |
| **Type of setting** | Rural | Rural | Urban | Rural |
| **Location** | East Mamprusi district | Migori County | Nairobi and Kiambu Counties | 20 districts in Uttar Pradesh |
| **Interview location** | Health facilities and respondents’ homes | Health facilities and respondents’ homes | Health facilities | Health facilities |
| **Time of interview after delivery** | Within 8 weeks of delivery | Within 9 weeks of delivery | Within 1 week of delivery | Within 48 hours of delivery |
| **Available languages** | Mampruli and Kokomba | English, Kiswahili, and Luo | English and Kiswahili | Hindi |
| **Period of Data Collection** | Mar – Apr 2017 | Aug – Sep 2016 | Aug – Dec 2016 | Aug – Oct 2017 |
| **Ethics Approval** | UCSF and NHRC ethical-review units | UCSF and KEMRI ethical-review units | UCSF and KEMRI ethical-review units | UCSF and CEL ethical-review units |
| **No. of experience of care indicators identified and analyzed** | 38 | 38 | 38 | 31 |
| **Self-reported Obstetric complication** | Available in dataset | Available in dataset | Available in dataset | Available in dataset |
| **Self-reported Severity of complication** | Available in dataset | Available in dataset |  |  |
| **Mode of Delivery (Self-reported C-section)** | Available in dataset | Available in dataset |  | Available in dataset |

Table adapted from previous publication ^1^

UCSF=University of California, San Francisco. KEMRI=Kenya Medical Research Institute (Kenya). IPA=Innovations for Poverty Action (Kenya). NHRC=Navrongo Health Research Center (Ghana). CEL=Community Empowerment Lab (India)

Blank spaces indicate missing in dataset

*Data available but missingness more than 50%

1 Afulani PA, Phillips B, Aborigo RA, Moyer CA. Person-centred maternity care in low-income and middle-income countries: analysis of data from Kenya, Ghana, and India. *The Lancet Global Health* 2019;**7**:e96–109. https://doi.org/10.1016/S2214-109X(18)30403-0.

**Appendix S2.**

Survey questions capturing reported obstetric complications

1. At any time during labor, delivery, or after delivery did you suffer from any health problems? 0 No

1 Yes

1. (IF YES) What problems did you have? Anything else?

Frequency of reported obstetric complications by country

| **Reported Delivery complication** | **Ghana,**  **n = 531** | **Rural Kenya,**  **n =873** | **Urban Kenya, n=531** | **India, n=2018** |
| --- | --- | --- | --- | --- |
| High Blood Pressure | 14 (3%) | 2 (0.2%) | 3 (0.6%) | 1 (0.1%) |
| Headache | 32 (6%) | 30 (3%) | 0 (0%) | 15 (0.7%) |
| Blurry Vision | 24 (5%) | 4 (0.5%) | 1 (0.2%) | 18 (0.9%) |
| Edema | 8 (2%) | 4 (0.5%) | 1 (0.2%) | 3 (0.2%) |
| Pre-Eclampsia | 0 (0%) | 3 (0.3%) | 1 (0.2%) |  |
| Convulsions/Eclampsia | 2 (0.4%) | 1 (0.1%) | 0 (0%) | 1 (0.1%) |
| Diabetes | 0 (0%) | 0 (0%) | 0 (0%) |  |
| Vaginal Bleeding | 15 (3%) | 32 (4%) | 16 (3%) | 11 (0.6%) |
| Foul-Smelling Vaginal Discharge | 0 (0%) | 1 (0.1%) | 0 (0%) | 4 (0.2%) |
| Lower Abdominal Pain | 75 (14%) | 57 (7%) | 1 (0.2%) | 88 (4%) |
| Urinary Tract Infection | 6 (1%) | 0 (0%) | 0 (0%) |  |
| Malaria | 19 (4%) | 19 (2%) | 0 (0%) |  |
| Fever | 4 (0.8%) | 3 (0.3%) | 0 (0%) | 34 (2%) |
| Excessive Vomiting | 3 ( 0.6%) | 2 (0.2) | 0 (0%) | 19 (0.9%) |
| Dizziness | 23 (4%) | 6 (0.7%) | 1 (0.2%) | 54 (3%) |
| Palpitations | 12 (2%) | 0 (0%) | 0 (0%) |  |
| Anemia | 8 (2%) | 4 (0.5%) | 0 (0%) | 10 (0.5%) |
| Slow or No Baby Movement | 5 (0.9%) | 1 (0.1%) | 9 (2%) |  |
| Prolonged/ Obstructed Labor | 28 (5%) | 22 (3%) | 32 (6%) | 0 (0%) |
| Baby’s Hands/Feet Came Out First | 2 (0.4%) | 5 (0.6%) | 5 (0.9%) |  |
| Torn Uterus | 1 (0.2%) | 2 (0.2%) | 2 (0.4%) | 4 (0.2%) |
| Fistula | 13 (2%) | 0 (0%) | 0 (0%) |  |
| Tetanus | 0 (0%) | 0 (0%) | 0 (0%) |  |
| Placenta Previa |  |  | 2 (0.4%) |  |
| Fast Breathing |  |  |  | 29 (1.44%) |
| Retained Placenta |  |  |  | 3 (0.15%) |
| Uterine Inversion |  |  |  | 2 (0.1%) |
| Reported obstetric complication overall | 189 (36%) | 157 (18%) | 70 (13%) | 229 (11%) |
| Notes: data not available in blank spaces | | | | |

**Question capturing severity of obstetric complication**

1. Will you say this problem was severe?

0 No

1 Yes

Question capturing c-section

Was your baby delivery by caesarean section?

0 No

1 Yes

**Appendix S3**.

| **Table 1: Characteristics of women from rural Kenya, urban Kenya, Ghana and India, N=3,953** | | | | |
| --- | --- | --- | --- | --- |
|  | **Rural Kenya,**  **n = 873** | **Urban Kenya**  **n = 531** | **Ghana**  **n = 531** | **India**  **n = 2018** |
| **Age** | N (%) | N (%) | N (%) | N (%) |
| 15 to 22 years | 354 (41%) | 159 (30%) | 127 (24%) | 659 (33%) |
| 23 to 25 years | 146 (17%) | 125 (24%) | 97 (18%) | 595 (29%) |
| 26 to 28 years | 140 (16%) | 114 (21%) | 67 (13%) | 427 (21%) |
| 29 to 48 years | 233 (27%) | 133 (25%) | 239 (45%) | 337 (17%) |
| **Parity (No. of prior births)** |  |  |  |  |
| 0-1 | 295 (34%) | 180 (34%) | 137 (26%) | 0 (0%) |
| 2 | 181 (21%) | 202 (38%) | 109 (21%) | 609 (46%) |
| 3 | 161 (18%) | 101 (19%) | 93 (18%) | 393 (30%) |
| 4+ | 235 (27%) | 48 (9%) | 189 (36%) | 310 (24%) |
| **Marital Status** |  |  |  |  |
| Single | 140 (16%) | 61 (11%) | 10 (2%) | 0 (0%) |
| Partnered/Cohabiting | 3 (0.3%) | 75 (14%) | 31 (6%) | 0 (0%) |
| Married | 635 (78%) | 383 (72%) | 488 (92%) | 2013 (100%) |
| Widowed/ Divorced/Separated | 45 (5%) | 12 (2%) | 2 (0.4%) | 5 (0.5%) |
| **Education** |  |  |  |  |
| No school or primary | 489 (56%) | 204 (38%) | 362 (68%) | 1328 (66%) |
| Post primary, vocational, or secondary | 273 (31%) | 242 (46%) | 153 (29%) | 441 (22%) |
| University/ College or above | 111 (13%) | 85 (16%) | 16 (3%) | 249 (12%) |
| **Employed** |  |  |  |  |
| No | 657 (75%) | 251 (47%) | 494 (93%) | 1905 (94%) |
| Yes | 216 (25%) | 280 (53%) | 36 (7%) | 113 (6%) |
| **Household wealth** |  |  |  |  |
| Poorest | 188 (22%) | 0 (0%) | 135 (26%) | 404 (20%) |
| Poor | 188 (22%) | 1 (0.2%) | 163 (31%) | 404 (20%) |
| Middle | 135 (16%) | 18 (3%) | 182 (35%) | 403 (20%) |
| Rich | 171 (20%) | 88 (17%) | 31 (6%) | 404 (20%) |
| Richest | 187 (22%) | 424 (80%) | 9 (2%) | 403 (20%) |
| **Had pregnancy complications** |  |  |  |  |
| No | 492 (56%) | 447 (84%) | 234 (44%) | 1593 (79%) |
| Yes | 381 (44%) | 84 (16%) | 297 (56%) | 425 (21%) |
| **Facility type** |  |  |  |  |
| Government Hospital | 398 (46%) | 432 (81%) | 60 (11%) | 703 (35%) |
| Government Health Center | 361 (41%) | 99 (19%) | 245 (47%) | 1315 (65%) |
| Mission or private facility | 111 (13%) | 0 (0%) | 220 (42%) | 0 (0%) |
| **Delivery provider gender** |  |  |  |  |
| Male | 326 (37%) | 74 (14%) | 42 (8%) | 9 (1%) |
| Female | 512 (59%) | 371 (70%) | 484 (91%) | 2001 (100%) |
| Both | 34 (4%) | 86 (16%) | 4 (1%) | 0 (0%) |
| **Obstetric Complications** |  |  |  |  |
| No obstetric complications | 716 (82%) | 461 (87%) | 342 (64%) | 1789 (89%) |
| Obstetric complications | 157 (18%) | 70 (13%) | 189 (36%) | 229 (11%) |
| **Mode of Delivery** |  |  |  |  |
| Vaginal delivery | 797 (92%) | -- | 483 (91%) | 2007 (99%) |
| C-section | 74 (8%) | -- | 47 (9%) | 11 (1%) |
| ***Duration in facility before delivery** |  |  |  |  |
| <2 hours | 366 (42%) | 163 (31%) | 202 (38%) | 863 (43%) |
| 2-5 hours | 299 (34%) | 82 (16%) | 188 (36%) | 624 (31%) |
| 6+ hours | 207 (24%) | 282 (54%) | 138 (26%) | 531 (26%) |
| *Notes: Table adapted from previous publication*^1^  ** Total duration in facility before and after delivery in India only* | | | | |

**Appendix S4.**

| **Distribution of experience of care indicators in four datasets, N = 3,953** | | | | | |
| --- | --- | --- | --- | --- | --- |
|  | **Ghana (n=531)** | **Rural Kenya (n=873)** | **Urban Kenya**  **(n =531)** | **India (n=2,018)** | **Pooled**  **(n=3,953)** |
| **Supportive Care Domain** | | | | | |
| **Time to care:** How did you feel about the amount of time you waited? Would you say it was | | | | | |
| Very long | 40 (8%) | 55 (6%) | 44 (8%) | 84 (4%) | 223 (6%) |
| Somewhat long | 59 (11%) | 88 (10%) | 141 (27%) | 177 (9%) | 465 (12%) |
| Somewhat short | 197 (37%) | 200 (23%) | 250 (47%) | 410 (20%) | 1057 (27%) |
| Very short | 235 (44%) | 530 (61%) | 96 (18%) | 1347 (67%) | 2208 (56%) |
| **Labor support:** Were you allowed to have someone you wanted (from outside of staff at the facility, such as family or friends) to stay with you during labor? | | | | | |
| No, never | 97 (18%) | 166 (19%) | 243 (46%) | 157 (8%) | 663 (17%) |
| Yes, a few times | 116 (22%) | 108 (12%) | 17 (3%) | 92 (5%) | 333 (8%) |
| Yes, most of the time | 98 (19%) | 232 (27%) | 9 (2%) | 295 (15%) | 634 (16%) |
| Yes, all the time | 220 (41%) | 367 (42%) | 262 (49%) | 1474 (73%) | 2323 (59%) |
| **Delivery support:** Were you allowed to have someone you wanted to stay with you during delivery? | | | | | |
| No, never | 379 (71%) | 532 (61%) | 241 (45%) | 175 (9%) | 1327 (34%) |
| Yes, a few times | 72 (14%) | 75 (9%) | 10 (2%) | 79 (4%) | 236 (6%) |
| Yes, most of the time | 50 (9%) | 111 (13%) | 2 (0.4%) | 270 (13%) | 433 (11%) |
| Yes, all the time | 30 (6%) | 155 (18%) | 278 (52%) | 1494 (74%) | 1957 (50%) |
| **Talked about feeling:** Did the doctors and nurses at the facility talk to you about how you were feeling? | | | | | |
| No, never | 133 (25%) | 119 (14%) | 100 (19%) | 817 (41%) | 1169 (30%) |
| Yes, a few times | 163 (31%) | 262 (30%) | 110 (21%) | 776 (38%) | 1311 (33%) |
| Yes, most of the time | 130 (24%) | 224 (26%) | 193 (36%) | 326 (16%) | 873 (22%) |
| Yes, all the time | 105 (20%) | 268 (30%) | 128 (24%) | 99 (5%) | 600 (15%) |
| **Received support when anxious:** Did the doctors, nurses or other staff at the facility try to understand your anxieties and fears? | | | | | |
| No, never | 119 (22%) | 203 (23%) | 131 (25%) | 456 (23%) | 909 (23%) |
| Yes, a few times | 161 (30%) | 203 (23%) | 59 (11%) | 667 (33%) | 1090 (28%) |
| Yes, most of the time | 90 (17%) | 151 (17%) | 86 (16%) | 442 (22%) | 769 (20%) |
| Yes, all the time | 161 (30%) | 316 (36%) | 255 (48%) | 453 (22%) | 1185 (30%) |
| **Attention when need help:** When you needed help, did you feel the doctors, nurses or other staff at the facility paid attention? | | | | | |
| No, never | 57 (11%) | 42 (5%) | 28 (5%) | 80 (4%) | 207 (5%) |
| Yes, a few times | 121 (23%) | 123 (14%) | 99 (19%) | 403 (20%) | 746 (19) |
| Yes, most of the time | 151 (28%) | 331 (38%) | 246 (46%) | 634 (31%) | 1362 (35%) |
| Yes, all the time | 202 (38%) | 377 (43%) | 158 (30%) | 901 (45%) | 1638 (41%) |
| **Took best care:** Did you feel the doctors, nurses or other staff at the facility took the best care of you? | | | | | |
| No, never | 16 (3%) | 22 (3%) | 6 (1%) | 69 (3%) | 113 (3%) |
| Yes, a few times | 66 (12%) | 84 (10%) | 76 (14%) | 444 (22%) | 670 (17%) |
| Yes, most of the time | 216 (41%) | 310 (36%) | 239 (45%) | 831 (41%) | 1596 (40%) |
| Yes, all the time | 233 (44%) | 457 (52%) | 210 (40%) | 674 (33%) | 1574 (40%) |
| **Control pain:** Do you feel the doctors or nurses did everything they could to help control your pain? | | | | | |
| No, never | 93 (18%) | 333 (38%) | 220 (41%) | 182 (9%) | 828 (21%) |
| Yes, a few times | 133 (25%) | 136 (16%) | 80 (15%) | 478 (24%) | 827 (21%) |
| Yes, most of the time | 137 (26%) | 191 (22%) | 132 (25%) | 759 (38%) | 1219 (31%) |
| Yes, all the time | 168 (32%) | 213 (24%) | 99 (19%) | 599 (30%) | 1079 (27%) |
| **Trust:** Did you feel you could completely trust the doctors, nurses, or other staff at the facility with regards to your care? | | | | | |
| No, never | 14 (3%) | 29 (3%) | 12 (2%) | 55 (3%) | 110 (3%) |
| Yes, a few times | 71 (13%) | 89 (10%) | 60 (11%) | 144 (7%) | 364 (9%) |
| Yes, most of the time | 190 (36%) | 297 (34%) | 171 (32%) | 453 (22%) | 1111 (28%) |
| Yes, all the time | 256 (48%) | 458 (53%) | 288 (54%) | 1366 (68%) | 2368 (60%) |
| **Enough staff present:** Do you think there was enough health staff in the facility to care for you? | | | | | |
| No, never | 22 (4%) | 116 (13%) | 58 (11%) | 36 (2%) | 232 (6%) |
| Yes, a few times | 137 (26%) | 122 (14%) | 77 (15%) | 320 (16%) | 656 (17%) |
| Yes, most of the time | 144 (27%) | 254 (29%) | 199 (38%) | 696 (35%) | 1293 (33%) |
| Yes, all the time | 228 (43%) | 381 (44%) | 197 (37%) | 966 (48%) | 1772 (45%) |
| **Crowded:** Thinking about the labor and postnatal wards, did you feel the health facility was crowded? | | | | | |
| Yes, all the time | 99 (19%) | 142 (16%) | 128 (24%) | 225 (11%) | 594 (15%) |
| Yes, most of the time | 84 (16%) | 144 (17%) | 67 (13%) | 563 (28%) | 858 (22%) |
| Yes, a few times | 228 (43%) | 182 (21%) | 51 (10%) | 803 (40%) | 1264 (32%) |
| No, never | 120 (23%) | 405 (46%) | 285 (54%) | 427 (21%) | 1237 (31%) |
| **Clean environment:** Thinking about the wards, washrooms, and the general environment of the health facility, will you say the facility was | | | | | |
| Very dirty | 2 (0.4%) | 9 (1%) | 3 (0.6%) | 355 (18%) | 357 (9%) |
| Dirty | 16 (3%) | 104 (12%) | 28 (5%) | 386 (19%) | 504 (13%) |
| Clean | 272 (51%) | 613 (70%) | 398 (75%) | 118 (6%) | 1661 (42%) |
| Very clean | 241 (45%) | 147 (17%) | 102 (19%) | 1159 (57%) | 1431 (36%) |
| **Water present at facility:** Was there water in the facility? | | | | | |
| No, never | 7 (1%) | 39 (5%) | 7 (1%) | 264 (13%) | 317 (8%) |
| Yes, a few times | 74 (14%) | 69 (8%) | 33 (6%) | 48 (2%) | 224 (6%) |
| Yes, most of the time | 151 (28%) | 226 (26%) | 142 (27%) | 222 (11%) | 741 (19%) |
| Yes, all the time | 299 (56%) | 539 (62%) | 349 (66%) | 1484 (74%) | 2671 (68%) |
| **Electricity present at facility:** Was there electricity in the facility? | | | | | |
| No, never | 1 (0.2%) | 49 (6%) | 0 (0%) | 8 (0.4%) | 58 (2%) |
| Yes, a few times | 27 (5%) | 73 (8%) | 15 (3%) | 135 (7%) | 250 (6%) |
| Yes, most of the time | 103 (19%) | 256 (29%) | 80 (15%) | 920 (46%) | 1359 (34%) |
| Yes, all the time | 400 (75%) | 495 (57%) | 436 (82%) | 955 (47%) | 2286 (58%) |
| **Felt safe at facility:** In general, did you feel safe in the health facility? | | | | | |
| No, never | 17 (3%) | 16 (2%) | 1 (0.2%) | 30 (2%) | 64 (2%) |
| Yes, a few times | 43 (8%) | 57 (7%) | 34 (6%) | 45 (2%) | 179 (5%) |
| Yes, most of the time | 188 (35%) | 198 (23%) | 91 (17%) | 226 (11%) | 703 (18%) |
| Yes, all the time | 283 (53%) | 602 (69%) | 405 (76%) | 1717 (85%) | 3007 (76%) |
| **Communication and Autonomy Domain** | | | | | |
| **Introduce self:** During your time in the health facility did the doctors, nurses, or other health care providers introduce themselves to you when they first came to see you? | | | | | |
| No, none of them | 378 (71%) | 671 (77%) | 452 (85%) | 1980 (98%) | 3481 (88%) |
| Yes, a few of them | 69 (13%) | 109 (13%) | 42 (8%) | 35 (2%) | 255 (7%) |
| Yes, most of them | 56 (11%) | 41 (5%) | 27 (5%) | 2 (0.1%) | 126 (3%) |
| Yes, all of them | 28 (5%) | 52 (6%) | 10 (2%) | 1 (0%) | 91 (2%) |
| **Called by name:** Did the doctors, nurses, or other health care providers call you by your name? | | | | | |
| No, never | 156 (29%) | 238 (27%) | 231 (44%) | 567 (28%) | 1192 (30%) |
| Yes, a few times | 118 (22%) | 171 (20%) | 132 (25%) | 436 (22%) | 857 (22%) |
| Yes, most of the time | 86 (16%) | 137 (16%) | 94 (18%) | 371 (18%) | 688 (17%) |
| Yes, all the time | 171 (32%) | 327 (38%) | 74 (14%) | 644 (32%) | 1216 (31%) |
| **Involvement in care:** Did you feel like the doctors, nurses or other staff at the facility involved you in decisions about your care? | | | | | |
| No, never | 139 (26%) | 169 (19%) | 43 (8%) | 1131 (56) | 1482 (38%) |
| Yes, a few times | 152 (29%) | 115 (13%) | 48 (9%) | 311 (15%) | 626 (16%) |
| Yes, most of the time | 115 (22%) | 172 (20%) | 66 (12%) | 255 (13%) | 608 (15%) |
| Yes, all the time | 125 (24%) | 417 (48%) | 374 (70%) | 321 (16%) | 1237 (31%) |
| **Consent to procedures:** Did the doctors, nurses or other staff at the facility ask your permission/consent before doing procedures on you? | | | | | |
| No, never | 127 (24%) | 314 (36%) | 197 (37%) | 1475 (73%) | 2113 (54%) |
| Yes, a few times | 88 (17%) | 119 (14%) | 80 (15%) | 282 (14%) | 569 (14%) |
| Yes, most of the time | 107 (20%) | 198 (23%) | 130 (25%) | 172 (9%) | 607 (15%) |
| Yes, all the time | 209 (39%) | 242 (28%) | 124 (23%) | 89 (4%) | 664 (17%) |
| **Delivery position choice:** During the delivery, do you feel like you were able to be in the position of your choice? | | | | | |
| No, never | 222 (42%) | 610 (70%) | 210 (40%) | 360 (18%) | 1402 (36%) |
| Yes, for a short time | 94 (18%) | 110 (13%) | 46 (9%) | 655 (33%) | 905 (21%) |
| Yes, most of the time | 127 (24%) | 74 (9%) | 86 (16%) | 418 (21%) | 705 (18%) |
| Yes, all the time | 88 (17%) | 79 (9%) | 189 (36%) | 585 (29%) | 941 (24%) |
| **Language:** Did the doctors, nurses or other staff at the facility speak to you in a language you could understand? | | | | | |
| No, never | 16 (3%) | 22 (3%) | 1 (0.2%) | 16 (1%) | 55 (1%) |
| Yes, a few times | 46 (9%) | 68 (8%) | 7 (1%) | 315 (16%) | 436 (11%) |
| Yes, most of the time | 141 (27%) | 185 (21%) | 58 (11%) | 1556 (77%) | 1940 (49%) |
| Yes, all the time | 328 (62%) | 598 (69%) | 465 (88%) | 131 (7%) | 1522 (39%) |
| **Explain exams/ procedures:** Did the doctors and nurses explain to you why they were doing examinations or procedures on you? | | | | | |
| No, never | 205 (39%) | 241 (28%) | 107 (20%) | 1393 (69%) | 1946 (49%) |
| Yes, a few times | 92 (17%) | 123 (14%) | 83 (16%) | 344 (17%) | 642 (16%) |
| Yes, most of the time | 114 (22%) | 218 (25%) | 127 (24%) | 174 (9%) | 633 (16%) |
| Yes, all the time | 120 (23%) | 291 (33%) | 214 (40%) | 107 (5%) | 732 (19%) |
| **Explain medicines:** Did the doctors and nurses explain to you why they were giving you any medicine? | | | | | |
| No, never | 193 (36%) | 151 (17%) | 95 (18%) | 1162 (58%) | 1820 (46%) |
| Yes, a few times | 106 (20%) | 106 (12%) | 68 (13%) | 400 (20%) | 680 (17%) |
| Yes, most of the time | 87 (16%) | 211 (24%) | 58 (11%) | 242 (12%) | 598 (15%) |
| Yes, all the time | 145 (27%) | 405 (46%) | 310 (58%) | 214 (11%) | 855 (22%) |
| **Able to ask questions:** Did you feel you could ask the doctors, nurses, or other staff at the facility any questions you had? | | | | | |
| No, never | 188 (35%) | 200 (23%) | 102 (19%) | 265 (13%) | 755 (19%) |
| Yes, a few times | 132 (25%) | 209 (24%) | 91 (17%) | 437 (22%) | 869 (22%) |
| Yes, most of the time | 120 (23%) | 185 (21%) | 159 (30%) | 543 (27%) | 1007 (26%) |
| Yes, all the time | 91 (17%) | 279 (32%) | 179 (34%) | 773 (38%) | 1322 (33%) |
| **Dignity and Respect Domain** | | | | | |
| **Treated with respect:** Did the doctors, nurses, or other staff at the facility treat you with respect? | | | | | |
| No, never | 24 (5%) | 19 (2%) | 12 (2%) | 143 (7%) | 198 (5%) |
| Yes, a few times | 98 (19%) | 78 (9%) | 65 (12%) | 299 (15%) | 540 (14%) |
| Yes, most of the time | 179 (34%) | 256 (29%) | 151 (28%) | 531 (26%) | 1117 (28%) |
| Yes, all the time | 230 (43%) | 520 (60%) | 303 (57%) | 1045 (52%) | 2098 (53%) |
| **Health staff Friendly:** Did the doctors, nurses, and other staff at the facility treat you in a friendly manner? | | | | | |
| No, never | 22 (4.1) | 26 (3) | 23 (4.3) | 92 (4.6) | 163 (4.1) |
| Yes, a few times | 102 (19.2) | 99 (11.3) | 66 (12.4) | 358 (17.7) | 625 (15.8) |
| Yes, most of the time | 180 (33.9) | 242 (27.7) | 187 (35.2) | 545 (27) | 1154 (29.2) |
| Yes, all the time | 227 (42.7) | 506 (58) | 255 (48) | 1023 (50.7) | 2011 (50.9) |
| **Verbal abuse:** Did you feel the doctors, nurses, or other health providers shouted at you, scolded, insulted, threatened, or talked to you rudely? | | | | | |
| Yes, many times | 31 (6%) | 17 (2%) | 10 (2%) | 14 (1%) | 72 (2%) |
| Yes, a few times | 28 (5%) | 24 (3%) | 0 (0%) | 131 (7%) | 183 (5%) |
| Yes, once | 38 (7%) | 58 (7%) | 86 (16%) | 212 (11%) | 394 (10%) |
| No, never | 434 (82%) | 774 (89%) | 435 (82%) | 1661 (82%) | 3304 (84%) |
| **Physical abuse:** Did you feel like you were treated roughly like pushed, beaten, slapped, pinched, physically restrained, or gagged? | | | | | |
| Yes, many times | 8 (2%) | 5 (0.6%) | 0 (0%) | 3 (0.1%) | 16 (0.4%) |
| Yes, a few times | 18 (3%) | 9 (1%) | 0 (0%) | 17 (1%) | 44 (1%) |
| Yes, once | 17 (3%) | 24 (3%) | 10 (2%) | 31 (2%) | 82 (2%) |
| No, never | 488 (92%) | 835 (96%) | 521 (98%) | 1967 (97%) | 3811 (96%) |
| **Visual privacy:** During examinations in the labor room, were you covered up with a cloth or blanket or screened with a curtain so that you did not feel exposed? | | | | | |
| No, never | 15 (3%) | 176 (20%) | 205 (39%) | 526 (26%) | 922 (23%) |
| Yes, a few times | 28 (5%) | 62 (7%) | 46 (9%) | 115 (6%) | 251 (6%) |
| Yes, most of the time | 116 (22%) | 113 (13%) | 55 (10%) | 228 (11%) | 512 (13%) |
| Yes, all the time | 372 (70%) | 522 (60%) | 225 (42%) | 1149 (57%) | 2268 (57%) |
| **Record confidentiality:** Do you feel like your health information was or will be kept confidential at this facility? | | | | | |
| No, never | 13 (2%) | 50 (6%) | 21 (4%) | 324 (16%) | 408 (10%) |
| Yes, a few times | 60 (11%) | 110 (13%) | 58 (11%) | 444 (22%) | 672 (17%) |
| Yes, most of the time | 124 (23%) | 269 (31%) | 132 (25%) | 387 (19%) | 912 (23%) |
| Yes, all the time | 334 (63%) | 444 (51%) | 320 (60%) | 863 (43%) | 1961 (50%) |
| **Additional Indicators** |  |  |  |  |  |
| **Bribe:** During your time at the facility, did any staff at the facility ask you or your family for "something" other than the official payment? | | | | | |
| Yes, all the time | 11 (2%) | 4 (0.5%) | 1 (0.2%) | 8 (0.4%) | 24 (0.6%) |
| Yes, most of the time | 7 (1%) | 9 (1%) | 0 (0%) | 156 (8%) | 172 (4%) |
| Yes, a few times | 33 (6%) | 57 (7%) | 0 (0%) | 526 (26%) | 616 (16%) |
| No, never | 480 (90%) | 803 (92%) | 530 (100%) | 1328 (66%) | 3141 (80%) |
| **Providers showed care:** Did the doctors, nurses, and other staff at the facility show that they cared about you? | | | | | |
| No, never | 19 (4%) | 29 (3%) | 9 (2%) |  |  |
| Yes, a few times | 80 (15%) | 75 (9%) | 80 (15%) |  |  |
| Yes, most of the time | 210 (40%) | 259 (30%) | 205 (39%) |  |  |
| Yes, all the time | 222 (42%) | 510 (58%) | 237 (45%) |  |  |
| **Privacy during discussions:** When you were speaking to the doctors, nurses, or other staff at the facility, did you feel other people not involved in your care could hear what you were discussing? | | | | | |
| Yes, all the time | 112 (21%) | 78 (9%) | 34 (6%) |  |  |
| Yes, most of the time | 103 (19%) | 87 (10%) | 41 (8%) |  |  |
| Yes, a few times | 82 (15%) | 153 (18%) | 42 (8%) |  |  |
| No, never | 234 (44%) | 555 (64%) | 414 (78%) |  |  |
| **Providers ask about pain:** Did the doctors and nurses ask how much pain you were in? | | | | | |
| No, never | 122 (23%) | 216 (25%) | 136 (26%) |  |  |
| Yes, a few times | 134 (25%) | 216 (25%) | 117 (22%) |  |  |
| Yes, most of the time | 121 (23%) | 207 (24%) | 162 (31%) |  |  |
| Yes, all the time | 154 (29%) | 234 (27%) | 116 (22%) |  |  |
| **Received attention during stay at facility:** Did you feel the doctors and nurses paid attention to you during your stay in the facility? | | | | | |
| No, never | 33 (6%) | 32 (4%) | 30 (6%) |  |  |
| Yes, a few times | 106 (20%) | 133 (15%) | 101 (19%) |  |  |
| Yes, most of the time | 168 (32%) | 340 (39%) | 232 (44%) |  |  |
| Yes, all the time | 224 (42%) | 368 (42%) | 168 (32%) |  |  |
| **Allowed to eat & drink:** Were you allowed to eat or drink when you were hungry/thirsty? | | | | | |
| No, never | 33 (6%) | 340 (39%) | 26 (5%) |  |  |
| Yes, a few times | 107 (20%) | 228 (26%) | 47 (9%) |  |  |
| Yes, most of the time | 123 (23%) | 160 (18%) | 161 (30%) |  |  |
| Yes, all the time | 268 (50%) | 145 (17%) | 297 (56%) |  |  |
| **Patient forced to stay against will due to lack of pay:** Did you feel like you were forced to stay at the health facility against your will because you could not pay your bill? | | | | | |
| Yes, for about three days or more | 2 (0.4%) | 9 (1%) | 1 (0.2%) |  |  |
| Yes, for one to two days | 14 (3%) | 9 (1%) | 2 (0.4%) |  |  |
| Yes, for less than one day | 22 (4%) | 27 (3%) | 1 (0.2%) |  |  |
| No, never | 493 (93%) | 828 (95%) | 527 (99%) |  |  |
| **Patient treated differently because of personal attributes:** During your time in the health facility, will you say you were treated differently because of any personal attribute, like your age, marital status, number of children, your education, wealth, your connections with the facility, or something like that? | | | | | |
| Yes, all the time | 2 (0.4%) | 16 (2%) | 0 (0%) |  |  |
| Yes, most of the time | 8 (2%) | 19 (2%) | 0 (0%) |  |  |
| Yes, a few times | 25 (5%) | 23 (3%) | 9 (2%) |  |  |
| No, never | 496 (93%) | 815 (93%) | 522 (98%) |  |  |
| *Notes: Table adapted from previous publication ^5^* | | | | | |

**Appendix S5.**

| **2x2 table of mode of delivery by obstetric complications, N = 3419 (row totals)** | | | | |
| --- | --- | --- | --- | --- |
|  |  | Obstetric complications | |  |
|  |  | No obstetric complications, n (%) | Obstetric complications, n (%) | Total n |
| Mode of delivery | Vaginal delivery | 2773 (84.4%) | 514 (15.6%) | 3287 (100%) |
|  | Cesarean delivery | 72 (54.6%) | 60 (45.5%) | 132 (100%) |
| Total |  | 2845 (83.2%) | 574 (16.8%) | 3419 (100%) |

| **2x2 table of mode of delivery by obstetric complications, n = 3419 (column totals)** | | | | |
| --- | --- | --- | --- | --- |
|  |  | Obstetric complications | |  |
|  |  | No obstetric complications, n (%) | Obstetric complications, n (%) | Total n |
| Mode of delivery | Vaginal delivery | 2773 (97.5%) | 514 (89.6%) | 3287 (96.1%) |
|  | Cesarean delivery | 72 (2.5%) | 60 (10.5%) | 132 (3.9%) |
| Total |  | 2845 (100%) | 574 (100%) | 3419 (100%) |

**Appendix S6.**

| **Difference in mean scores of experience of care indicators by severe obstetric complications in Ghana and Rural Kenya, N=1,404** | | | | |
| --- | --- | --- | --- | --- |
|  | **Difference between mean scores of no complication and mild complication**  **(Mild – none)** | **Difference between mean scores of no complication and severe complication (Severe - none)** | **Difference between mean scores of mild and severe complications**  **(Severe-mild)** | **P-value** |
| **Supportive Care, mean (SD)** | **-1.8** | **-3.7** | **-1.9** | **0.00** |
| Time to care | 0.03 | -0.16 | -0.20 | **0.04** |
| Labor support | -0.13 | 0.08 | 0.21 | 0.28 |
| Delivery support | **-0.36** | **-0.31** | 0.04 | **0.00** |
| Talked about feeling | 0.05 | -0.07 | -0.12 | 0.55 |
| Support Anxiety | 0.02 | -0.13 | -0.15 | 0.28 |
| Attention when need help | 0.16 | -0.05 | -0.21 | 0.14 |
| Took best care | 0.05 | -0.10 | -0.15 | 0.19 |
| Control pain | **0.41** | 0.17 | -0.23 | **0.00** |
| Trust | 0.07 | -0.02 | -0.09 | 0.61 |
| Enough staff present | -0.04 | 0.04 | 0.08 | 0.77 |
| Crowded | -0.04 | -0.20 | -0.16 | 0.06 |
| Clean environment | -0.07 | 0.03 | 0.10 | 0.16 |
| Water at facility | -0.03 | -0.08 | -0.05 | 0.30 |
| Electricity at facility | 0.17 | 0.09 | -0.08 | **0.04** |
| Felt safe at facility | 0.06 | 0.03 | -0.03 | 0.64 |
| **Communication and Autonomy** |  |  |  | **0.00** |
| Introduce self | 0.00 | -0.12 | -0.12 | 0.15 |
| Called by name | -0.13 | 0.18 | **0.31** | 0.05 |
| Involvement in care | -0.15 | **-0.26** | -0.11 | **0.01** |
| Consent to procedures | **0.25** | -0.11 | **-0.35** | 0.05 |
| Delivery position choice | 0.17 | 0.03 | -0.14 | 0.28 |
| Language | 0.08 | 0.01 | -0.07 | 0.57 |
| Explain exams/ procedures | **-0.38** | -0.10 | **0.28** | **0.01** |
| Explain medicines | -0.12 | **-0.31** | -0.20 | **0.00** |
| Able to ask questions | -0.14 | -0.13 | 0.01 | 0.18 |
| **Dignity and Respect Domain** |  |  |  | **0.03** |
| Treated with respect | -0.21 | -0.02 | 0.19 | **0.03** |
| Friendly | -0.18 | -0.05 | 0.13 | 0.09 |
| Verbal abuse | -0.13 | -0.16 | -0.03 | **0.00** |
| Physical abuse | -0.04 | -0.11 | -0.06 | **0.00** |
| Visual privacy (were covered) | 0.16 | 0.05 | -0.11 | 0.30 |
| Record confidentiality | **0.31** | 0.15 | -0.16 | **0.00** |
| **Additional Experience of Care Indicators** | | | | |
| Bribe | -0.01 | -0.10 | -0.09 | **0.01** |
| Lack of Privacy during discussions | -0.06 | 0.12 | 0.18 | 0.24 |
| Providers ask about pain | 0.18 | 0.10 | -0.07 | 0.15 |
| attention during stay at facility | 0.11 | -0.07 | -0.18 | 0.19 |
| Allowed to eat & drink | 0.22 | **0.30** | 0.08 | **0.00** |
| Patient forced to stay | 0.02 | -0.01 | -0.03 | 0.81 |
| Patient treated differently | 0.05 | -0.07 | -0.12 | **0.04** |
| Providers showed care | -0.04 | 0.00 | 0.04 | 0.91 |
| *Blue highlight = statistically significant (p<0.05) differences and differ*  *Bolded values = greater than 0.25 difference* | | | | |

**Appendix S7**

| **Multivariate Linear regression of PCMC on selected covariates in Kenya, Ghana, and India** | | | | |
| --- | --- | --- | --- | --- |
|  | **Rural Kenya** | **Urban Kenya** | **Ghana** | **India** |
|  | Coef.  [robust std. Err] | Coef.  [robust std. Err] | Coef.  [robust std. Err] | Coef.  [robust std. Err] |
| **Obstetric complications** |  |  |  |  |
| No obstetric complications | Ref. |  |  |  |
| Obstetric complications | -3.24*  [-6.09, -0.40] | -1.05  [-4.58, 2.49] | -0.56  [-3.54, 2.41] | 0.87  [-1.45, 3.19] |
| **Mode of Delivery** |  |  |  |  |
| Vaginal delivery | Ref. |  |  |  |
| Cesarean delivery | 8.22***  [4.82, 11.6] | -- | -1.23  [-7.38, 4.92] | -7.57***  [-9.98, -5.16] |
| **^a^ Time spent in facility before delivery** |  |  |  |  |
| <2 hours | Ref |  |  |  |
| 2-5 hours | -4.89***  [-7.16, -2.63] | -1.42  [-5.00, 2.16] | -2.64  [-5.79, 0.51] | -1.49  [-3.09, 0.11] |
| 6+ hours | -5.61***  [-8.26, -2.96] | 1.18  [-1.20, 3.56] | 1.55  [-2.02, 5.12] | -2.44*  [-4.32, -0.56] |
| **Age** |  |  |  |  |
| 15 to 22 years | Ref. |  |  |  |
| 23 to 25 years | 0.12  [-3.01, 3.25] | 2.98  [-0.18, 6.15] | 0.39  [-4.96, 5.75] | 0.11  [-2.29, 2.51] |
| 26 to 28 years | -2.67  [-6.12, 0.79] | 0.46 [-3.24, 4.15] | -4.98  [-11.3, 1.31] | 1.33  [-1.21, 3.87] |
| 29 to 48 years | -0.21  [-3.78, 3.36] | 2.79  [-1.34, 6.92] | -1.89  [-7.76, 3.99] | 1.83  [-0.95, 4.61] |
| **Parity** |  |  |  |  |
| 0-1 | Ref. |  |  | -- |
| 2 | 0.65  [-2.49, 3.80] | -2.48  [-5.56, 0.59] | -1.65  [-7.19, 3.89] | Ref |
| 3 | 0.21  [-3.32, 3.74] | 1.63  [-2.54, 5.79] | 1.81  [-3.96, 7.58] | -0.39  [-2.20, 1.43] |
| 4+ | -1.84  [-5.63, 1.95] | 1.53  [-3.85, 6.91] | 2.09  [-4.16, 8.34] | -0.29  [-2.39, 1.81] |
| **Marital Status** |  |  |  | , |
| Single | Ref. |  |  | -- |
| Partnered/Cohabiting | -2.01  [-22.5, 18.5] | 13.0***  [8.76, 17.3] | 9.36  [-2.91, 21.6] | -- |
| Married | 3.10  [-0.27, 6.46] | 3.29  [-0.46, 7.03] | 7.50  [-2.78, 17.8] | Ref |
| Widowed/Divorced/Separated | -0.18  [-5.94, 5.59] | 5.36  [-2.19, 12.9] | 21.1**  [7.43, 34.7] | -0.36  [-12.8, 12.0] |
| **Education** |  |  |  |  |
| No school or primary | Ref. |  |  |  |
| Post primary/ vocational or secondary | -0.60  [-3.01, 1.81] | 2.41*  [0.0034, 4.83] | 2.71  [-1.06, 6.49] | 1.63  [-0.26, 3.52] |
| College or above | -0.94  [-4.64, 2.77] | 2.11  [-1.67, 5.89] | 6.51  [-3.54, 16.6] | 3.99***  [1.76, 6.21] |
| **Employed** |  |  |  |  |
| No | Ref |  |  |  |
| Yes | 6.16***  [3.60, 8.71] | -2.57*  [-4.87, -0.27] | -7.02*  [-12.7, -1.37] | 3.84*  [0.83, 6.84] |
| **Household Wealth** |  |  |  |  |
| Poorest/Poor | Ref. |  |  |  |
| Middle | 1.38  [-1.62, 4.38] | 7.90*  [0.012, 15.8] | 2.83  [-0.23, 5.90] | 2.50**  [0.70, 4.30] |
| Rich/Richest | 3.02*  [0.52, 5.52] | 4.53*  [0.079, 8.99] | 3.09  [-2.49, 8.67] | 0.98  [-0.68, 2.64] |
| **Pregnancy Complications** |  |  |  |  |
| No | Ref. |  |  |  |
| Yes | 2.53*  [0.43, 4.64] | -0.69  [-3.81, 2.42] | 4.49**  [1.64, 7.33] | -1.13  [-2.84, 0.58] |
| **Provider Sex** |  |  |  |  |
| Male | Ref. |  |  |  |
| Female | 0.18  [-1.85, 2.22] | -0.29 [-3.35, 2.77] | 0.71  [-5.67, 7.10] | -22.0***  [-24.2, -19.8] |
| Both | 8.28**  [3.29, 13.3] | -0.18  [-4.16, 3.80] | 1.73  [-23.3, 26.8] | -- |
| Constant | 62.7***  [58.9, 66.5] | 56.3***  [50.0, 62.5] | 52.4***  [39.7, 65.1] | 83.8***  [80.3, 87.3] |
| Observations | 865 | 527 | 514 | 1305 |
| *Notes: 95% confidence intervals in brackets*  ** p<0.05, ** p<0.01, *** p<0.001*  *-- data not available in dataset*  *a. Time in facility variable measured differently. Tine in facility before delivery was reported in rural Kenya, urban Kenya, and Ghana. Total time in facility reported for women in India* | | | | |

Appendix S8.

| **Multivariable logistic regression models of C-section on PCMC and PCMC domains (rural Kenya, Ghana and India), n =2687** | | |
| --- | --- | --- |
| **C-Section** | **Odds Ratio** | **Robust Std. Err** |
| PCMC | 1.02** | [1.01, 1.03] |
| Supportive Care | 1.01 | [0.99, 1.04] |
| Communication and Autonomy | 1.02** | [1.00, 1.03] |
| Dignity and Respect | 0.98* | [0.97, 1.00] |
| *Notes: does not include data from urban Kenya*  *Each row corresponds to individual models of PCMC, Supportive Care, Communication and autonomy, and Dignity and respect as independent variables*  *Controlling for age, parity, marital status, education, paid employment, household wealth, pregnancy complications, provider gender, country*  *Exponentiated coefficients; 95% confidence intervals in brackets*  ** p<0.05, ** p<0.01, *** p<0.001*  *Full table available upon request* | | |
